# Supplementary material for: All-dielectric chiral-field-enhanced Raman optical activity
Source: Nat Commun. 2021 May 24;12:3062. doi: 10.1038/s41467-021-23364-w (PMC8144208; doi:10.1038/s41467-021-23364-w)
Supplement: Supplementary file 1 — Supplementary Information [file 41467_2021_23364_MOESM1_ESM.pdf]

Supplementary Information

for

All-dielectric chiral-field-enhanced Raman optical activity

by Xiao et al.

Supplementary Note 1: Optically isotropic response of the silicon nanodisk array

The silicon nanodisk array can be expressed as a Jones matrix $\mathbf{R}$ in Cartesian coordinates as

$$\mathbf{R} = \begin{pmatrix} r_{\mathrm{xx}} & r_{\mathrm{xy}} \\ r_{\mathrm{yx}} & r_{\mathrm{yy}} \end{pmatrix}, \tag{1}$$

where the x-axis and y-axis are in the plane of the silicon nanodisk array while the z-axis is normal to it. The input state $\mathbf{I}$ and the output state $\mathbf{O}$ of light can be expressed in terms of certain x, y bases as

$$\mathbf{I} = \begin{pmatrix} i_{\mathrm{x}} \\ i_{\mathrm{y}} \end{pmatrix}, \mathbf{O} = \begin{pmatrix} o_{\mathrm{x}} \\ o_{\mathrm{y}} \end{pmatrix}. \tag{2}$$

Their relation is given by $\mathbf{O} = \mathbf{RI}$. Moreover, the C_4 operation matrix with respect to the z-axis can be expressed as

$$\mathbf{C} = \begin{pmatrix} \cos 2\pi/4 & \sin 2\pi/4 \\ -\sin 2\pi/4 & \cos 2\pi/4 \end{pmatrix}. \tag{3}$$

By applying $\mathbf{C}$ to the input and output states of light, we can obtain a new input state $\mathbf{I'} = \mathbf{CI}$ and a new output state $\mathbf{O'} = \mathbf{CO}$. As the nanodisk array satisfies the C_4 symmetry, the relation of $\mathbf{O'} = \mathbf{RI'}$ is satisfied for the new input and output states. Then, the relation of $\mathbf{CO} = \mathbf{RCI}$ can be deduced from the above equations. Further taking the relation of $\mathbf{O} = \mathbf{RI}$ into consideration, we can obtain $\mathbf{C}^{-1}\mathbf{RC} = \mathbf{R}$. By solving this matrix equation, we find $r_{xx} = r_{yy}$ and $r_{xy} = -r_{yx}$. Additionally, the silicon nanodisk array also satisfies the mirror symmetry with respect to the y-axis, such that its operation matrix can be expressed as

$$\boldsymbol{M}_{\mathrm{y}} = \begin{pmatrix} -1 & 0 \\ 0 & 1 \end{pmatrix}. \tag{4}$$

Similar to the deduction of the C_4 symmetry, we obtain $\mathbf{M_y}^{-1}\mathbf{RM_y} = \mathbf{R}$. By solving this matrix equation, we obtain $r_{\mathrm{xy}} = 0$ and $r_{\mathrm{yx}} = 0$. Taking all the above calculations into consideration, the Jones matrix $\mathbf{R}$ of the silicon nanodisk array can be rewritten as

$$\mathbf{R} = \begin{pmatrix} r_{\mathrm{xx}} & 0 \\ 0 & r_{\mathrm{xx}} \end{pmatrix}. \tag{5}$$

Therefore, the silicon nanodisk array is optically isotropic.

Supplementary Note 2: Dark mode excited in the silicon nanodisk array

We designed and simulated the dark mode excited in the silicon nanodisk array by using a commercial finite-element-method (FEM) software kit (COMSOL Multiphysics). We defined incident RCP or LCP light by introducing a 90˚ or -90˚ phase shift between the x-component and y-component of the electric field via port boundary conditions. Also, we used periodic boundary conditions to define an area as large as the unit cell of the silicon nanodisk array. The silicon nanodisk array was defined as being immersed in an alpha-pinene solution on a 2-μm-thick buried oxide layer on a silicon substrate. The refractive indices of the alpha-pinene solution and the buried-oxide layer were set to be 1.465 and 1.45, respectively. The refractive index of the silicon used in the simulation came from a report by Vuye et al.[1]. The simulated electric-field and magnetic-field distributions of the excited dark mode in the silicon nanodisk array at the wavelength of 532 nm are shown in Fig. 1f and Fig. 1e, respectively. The black arrows indicate the directions of the electric fields while the white arrows indicate the directions of the magnetic fields. It is evident in Fig. 1f that two electric-field vortices with opposite directions are generated in the nanodisk at a slice which is parallel to the nanodisk array. The electric-field vortices with opposite directions induce magnetic fields with opposite directions at a slice vertical to the nanodisk array as shown in Fig. 1e. The induced magnetic fields form a vortex that corresponds to a toroidal dipole. The destructive interference between the toroidal dipole and electric dipole induced in the nanodisk is the excited dark mode[2]. The dark mode partially cancels light scattering in the far field while it maintains the near-field excitation, which corresponds to the reflection valley and the near-field enhancement of the silicon nanodisk array as shown in Fig. 1a.

Supplementary Note 3: Chiral-field-enhanced ROA of chiral molecules

The electric dipole moment and magnetic dipole moment of a chiral molecule in an electromagnetic field is given by[3]

$$\mu_\alpha = \alpha_{\alpha\beta}E_\beta + \alpha'_{\alpha\beta}\dot{E}_\beta + \frac{1}{3}A_{\alpha,\beta\gamma}\nabla_\beta E_\gamma + \frac{1}{3}A'_{\alpha,\beta\gamma}\nabla_\beta \dot{E}_\gamma - iG_{\alpha\beta}B_\beta - iG'_{\alpha\beta}\dot{B}_\beta + \cdots, \quad (6)$$

$$m_{\alpha} = \chi_{\alpha\beta}B_{\beta} + \chi'_{\alpha\beta}\dot{B}_{\beta} + iG_{\alpha\beta}E_{\beta} + iG'_{\alpha\beta}\dot{E}_{\beta} + \frac{1}{3}D_{\alpha,\beta\gamma}\nabla_{\beta}E_{\gamma} + \frac{1}{3}D'_{\alpha,\beta\gamma}\nabla_{\beta}\dot{E}_{\gamma} + \cdots, \tag{7}$$

where $\alpha_{\alpha\beta}$, $\chi_{\alpha\beta}$, $A_{\alpha,\beta\gamma}$, $G_{\alpha\beta}$, $D_{\alpha,\beta\gamma}$, E_{β}, and B_{β} are the electric polarizability, magnetic susceptibility, electric dipole-electric quadrupole polarizability, electric dipole-magnetic dipole polarizability, magnetic dipole-electric quadrupole polarizability, electric field, and magnetic field, respectively. The Einstein notations are used to express the above equations. If we omit the high-order terms and rewrite the electric and magnetic dipole moments as complex vectors, Eqs. (6) and (7) can be written as

$$\tilde{\boldsymbol{\mu}} = \tilde{\alpha}\tilde{\mathbf{E}} + \frac{1}{3}\widetilde{A_{\gamma}}\boldsymbol{\nabla}\widetilde{E_{\gamma}} - i\tilde{G}\tilde{\mathbf{B}}, \tag{8}$$

$$\tilde{\mathbf{m}} = \tilde{\chi}\tilde{\mathbf{B}} + i\tilde{G}\tilde{\mathbf{E}}. \tag{9}$$

As Raman scattering consists of two processes, namely, the excitation process and the radiation process, the electric and magnetic dipole moments that correspond to the above two processes can be described as follows. For the excitation process, the electric and magnetic dipole moments can be written as

$$\widetilde{\boldsymbol{\mu}^{\mathrm{e}}} = \widetilde{\alpha^{\mathrm{e}}}\widetilde{\mathbf{E}^{\mathrm{e}}} + \frac{1}{3}\widetilde{A_{\gamma}^{\mathrm{e}}}\boldsymbol{\nabla}\widetilde{E_{\gamma}^{\mathrm{e}}} - i\widetilde{G^{\mathrm{e}}}\widetilde{\mathbf{B}^{\mathrm{e}}}, \tag{10}$$

$$\widetilde{\mathbf{m}^{\mathrm{e}}} = \widetilde{\chi^{\mathrm{e}}}\widetilde{\mathbf{B}^{\mathrm{e}}} + i\widetilde{G^{\mathrm{e}}}\widetilde{\mathbf{E}^{\mathrm{e}}}, \tag{11}$$

where the superscript e indicates the physical variables of the excitation process. For the excitation rate R^{e} of Raman scattering, we have

$$R^{\mathrm{e}} \propto \langle \mathbf{E}^{\mathrm{e}} \cdot \dot{\boldsymbol{\mu}}^{\mathrm{e}} + \mathbf{B}^{\mathrm{e}} \cdot \dot{\mathbf{m}}^{\mathrm{e}} \rangle = \frac{\omega^{\mathrm{e}}}{2}\left(\widehat{\widetilde{\mathbf{E}^{\mathrm{e}}}^{*} \cdot \widetilde{\boldsymbol{\mu}^{\mathrm{e}}}} + \widehat{\widetilde{\mathbf{B}^{\mathrm{e}}}^{*} \cdot \widetilde{\mathbf{m}^{\mathrm{e}}}}\right), \tag{12}$$

where the brackets indicate average values over time, $\mathbf{E}^{\mathrm{e}}$, $\mathbf{B}^{\mathrm{e}}$, $\boldsymbol{\mu}^{\mathrm{e}}$, and $\mathbf{m}^{\mathrm{e}}$ are the time-dependent real parts of $\widetilde{\mathbf{E}^{\mathrm{e}}}$, $\widetilde{\mathbf{B}^{\mathrm{e}}}$, $\widetilde{\boldsymbol{\mu}^{\mathrm{e}}}$, and $\widetilde{\mathbf{m}^{\mathrm{e}}}$, respectively, ω^{e} is the angular frequency, and the hat indicates the time-dependent imaginary part of each parameter. By substituting Eqs. (10) and (11) into Eq. (12), the excitation rate can be written as

$$R^{\mathrm{e}} \propto \frac{\omega^{\mathrm{e}}}{2}\left[\widehat{\alpha^{\mathrm{e}}}\left|\widetilde{\mathbf{E}^{\mathrm{e}}}\right|^{2} + \frac{1}{3}\widetilde{\mathbf{E}^{\mathrm{e}}}^{*} \cdot \widehat{A_{\gamma}^{\mathrm{e}}}\boldsymbol{\nabla}\widetilde{E_{\gamma}^{\mathrm{e}}} + \widehat{\chi^{\mathrm{e}}}\left|\widetilde{\mathbf{B}^{\mathrm{e}}}\right|^{2}\right] + \omega^{\mathrm{e}}\widehat{G^{\mathrm{e}}}\left(\widehat{\widetilde{\mathbf{E}^{\mathrm{e}}}^{*} \cdot \widetilde{\mathbf{B}^{\mathrm{e}}}}\right), \tag{13}$$

where $\widehat{\alpha^{\mathrm{e}}}$, $\widehat{A_{\gamma}^{\mathrm{e}}}$, $\widehat{\chi^{\mathrm{e}}}$ and $\widehat{G^{\mathrm{e}}}$ are the time-dependent imaginary parts of $\widetilde{\alpha^{\mathrm{e}}}$, $\widetilde{A_{\gamma}^{\mathrm{e}}}$, $\widetilde{\chi^{\mathrm{e}}}$ and $\widetilde{G^{\mathrm{e}}}$. Based on the definition of optical chirality, the optical chirality of the electromagnetic field in the excitation process C^{e} is given by

$$C^{\mathrm{e}} = \frac{\varepsilon_{0}}{2}\mathbf{E}^{\mathrm{e}} \cdot \boldsymbol{\nabla} \times \mathbf{E}^{\mathrm{e}} + \frac{1}{2\mu_{0}}\mathbf{B}^{\mathrm{e}} \cdot \boldsymbol{\nabla} \times \mathbf{B}^{\mathrm{e}}. \tag{14}$$

Using the Maxwell equations, $\nabla \times \mathbf{E}^{\mathrm{e}} = i\omega^{\mathrm{e}}\mathbf{B}^{\mathrm{e}}$ and $\nabla \times \mathbf{B}^{\mathrm{e}} = -i\omega^{\mathrm{e}}\mu_0\varepsilon_0\mathbf{E}^{\mathrm{e}}$, the optical chirality can be rewritten as

$$C^{\mathrm{e}} = \frac{\varepsilon_0}{2}\left(\dot{\mathbf{B}}^{\mathrm{e}} \cdot \mathbf{E}^{\mathrm{e}} - \dot{\mathbf{E}}^{\mathrm{e}} \cdot \mathbf{B}^{\mathrm{e}}\right) = \frac{\varepsilon_0\omega}{2}\left(\widehat{\widetilde{\mathbf{E}^{\mathrm{e}}}^{*} \cdot \widetilde{\mathbf{B}^{\mathrm{e}}}}\right). \tag{15}$$

By substituting Eq. (15) into Eq. (13), the excitation rate is found to be

$$R^{\mathrm{e}} \propto \frac{\omega^{\mathrm{e}}}{2}\left[\widehat{\alpha^{\mathrm{e}}}\left|\widetilde{\mathbf{E}^{\mathrm{e}}}\right|^2 + \frac{1}{3}\widetilde{\mathbf{E}^{\mathrm{e}}}^{*} \cdot \widehat{A_{\gamma}^{\mathrm{e}}}\nabla\widetilde{E_{\gamma}^{\mathrm{e}}} + \widehat{\chi^{\mathrm{e}}}\left|\widetilde{\mathbf{B}^{\mathrm{e}}}\right|^2\right] + \frac{2C^{\mathrm{e}}\widehat{G^{\mathrm{e}}}}{\varepsilon_0}. \tag{16}$$

If we express the middle term in the brackets as $\frac{1}{3}\widetilde{\mathbf{E}^{\mathrm{e}}}^{*} \cdot \widehat{A_{\gamma}^{\mathrm{e}}}\nabla\widetilde{E_{\gamma}^{\mathrm{e}}} = T_s^{\mathrm{e}} + T_{\mathrm{as}}^{\mathrm{e}}$, where T_s^{e} is the chirally symmetric part while $T_{\mathrm{as}}^{\mathrm{e}}$ is the chirally antisymmetric part, Eq. (16) can be expressed as

$$R^{\mathrm{e}} \propto \frac{\omega^{\mathrm{e}}}{2}\left(\widehat{\alpha^{\mathrm{e}}}\left|\widetilde{\mathbf{E}^{\mathrm{e}}}\right|^2 + T_{\mathrm{s}}^{\mathrm{e}} + \widehat{\chi^{\mathrm{e}}}\left|\widetilde{\mathbf{B}^{\mathrm{e}}}\right|^2\right) + \frac{\omega^{\mathrm{e}}T_{\mathrm{as}}^{\mathrm{e}}}{2} + \frac{2C^{\mathrm{e}}\widehat{G^{\mathrm{e}}}}{\varepsilon_0}, \tag{17}$$

where the first term on the right side is chirally symmetric while the last two terms are chirally antisymmetric. Thus, for a chiral molecule, its excitation rates with opposite optical chirality values can be expressed as

$$R_{+}^{\mathrm{e}} \propto \frac{\omega^{\mathrm{e}}}{2}\left(\widehat{\alpha^{\mathrm{e}}}\left|\widetilde{\mathbf{E}^{\mathrm{e}}}\right|^2 + T_{\mathrm{s}}^{\mathrm{e}} + \widehat{\chi^{\mathrm{e}}}\left|\widetilde{\mathbf{B}^{\mathrm{e}}}\right|^2\right) + \frac{\omega^{\mathrm{e}}T_{\mathrm{as}}^{\mathrm{e}}}{2} + \frac{2C^{\mathrm{e}}\widehat{G^{\mathrm{e}}}}{\varepsilon_0}, \tag{18}$$

$$R_{-}^{\mathrm{e}} \propto \frac{\omega^{\mathrm{e}}}{2}\left(\widehat{\alpha^{\mathrm{e}}}\left|\widetilde{\mathbf{E}^{\mathrm{e}}}\right|^2 + T_{\mathrm{s}}^{\mathrm{e}} + \widehat{\chi^{\mathrm{e}}}\left|\widetilde{\mathbf{B}^{\mathrm{e}}}\right|^2\right) - \frac{\omega^{\mathrm{e}}T_{\mathrm{as}}^{\mathrm{e}}}{2} - \frac{2C^{\mathrm{e}}\widehat{G^{\mathrm{e}}}}{\varepsilon_0}. \tag{19}$$

For the radiation process, the electric and magnetic dipole moments can be written as

$$\widetilde{\boldsymbol{\mu}^{\mathrm{r}}} = \widetilde{\alpha^{\mathrm{r}}}\widetilde{\mathbf{E}^{\mathrm{r}}} + \frac{1}{3}\widetilde{A_{\gamma}^{\mathrm{r}}}\nabla\widetilde{E_{\gamma}^{\mathrm{r}}} - i\widetilde{G^{\mathrm{r}}}\widetilde{\mathbf{B}^{\mathrm{r}}}, \tag{20}$$

$$\widetilde{\mathbf{m}^{\mathrm{r}}} = \widetilde{\chi^{\mathrm{r}}}\widetilde{\mathbf{B}^{\mathrm{r}}} + i\widetilde{G^{\mathrm{r}}}\widetilde{\mathbf{E}^{\mathrm{r}}}, \tag{21}$$

where the superscript r indicates the physical variables of the radiation process. For the radiation rate R^{r} of Raman scattering, we have

$$R^{\mathrm{r}} \propto \langle \mathbf{E}^{\mathrm{r}} \cdot \dot{\boldsymbol{\mu}}^{\mathrm{r}} + \mathbf{B}^{\mathrm{r}} \cdot \dot{\mathbf{m}}^{\mathrm{r}} \rangle = \frac{\omega^{\mathrm{r}}}{2}\left(\widehat{\widetilde{\mathbf{E}^{\mathrm{r}}}^{*} \cdot \widetilde{\boldsymbol{\mu}^{\mathrm{r}}} + \widetilde{\mathbf{B}^{\mathrm{r}}}^{*} \cdot \widetilde{\mathbf{m}^{\mathrm{r}}}}\right). \tag{22}$$

As Eq. (22) has an identical mathematical form to Eq. (12), we can obtain the radiation rate by following the similar deduction from Eq. (13) to Eq. (16). Then, the radiation rate can be expressed as

$$R^{\mathrm{r}} \propto \frac{\omega^{\mathrm{r}}}{2}\left(\widehat{\alpha^{\mathrm{r}}}\left|\widetilde{\mathbf{E}^{\mathrm{r}}}\right|^2 + \frac{1}{3}\widetilde{\mathbf{E}^{\mathrm{r}}}^{*} \cdot \widehat{A_{\gamma}^{\mathrm{r}}}\nabla\widetilde{E_{\gamma}^{\mathrm{r}}} + \widehat{\chi^{\mathrm{r}}}\left|\widetilde{\mathbf{B}^{\mathrm{r}}}\right|^2\right) + \frac{2C^{\mathrm{r}}\widehat{G^{\mathrm{r}}}}{\varepsilon_0}. \tag{23}$$

Since Raman scattering is a two-step process, it is necessary to define the radiation step based on the excitation step. For the excitation rate of R_{+}^{e}, we define C_{+}^{r} as the optical chirality of the radiation and define $\frac{1}{3}\widetilde{\mathbf{E}_{+}^{\mathrm{r}}}^{*} \cdot$

$\widehat{A^{\mathrm{r}}_{\gamma+}}\nabla\widetilde{E^{\mathrm{r}}_{\gamma+}} = T_{+}$ as the radiation contribution from the electric dipole-electric quadrupole polarizability. For the excitation rate of R^{e}_{-}, we define C^{r}_{-} as the optical chirality of the radiation and define $\frac{1}{3}\widetilde{\mathbf{E}^{\mathrm{r}}_{-}}^{*}\cdot\widehat{A^{\mathrm{r}}_{\gamma-}}\nabla\widetilde{E^{\mathrm{r}}_{\gamma-}} = T_{-}$ as the radiation contribution from the electric dipole-electric quadrupole polarizability. Thus, the radiation rates for the excitation rates of R^{e}_{+} and R^{e}_{-} can be written respectively as

$$R^{\mathrm{r}}_{+} \propto \frac{\omega^{\mathrm{r}}}{2}\left(\widehat{\alpha^{\mathrm{r}}}\left|\widetilde{\mathbf{E}^{\mathrm{r}}}\right|^{2} + T_{+} + \widehat{\chi^{\mathrm{r}}}\left|\widetilde{\mathbf{B}^{\mathrm{r}}}\right|^{2}\right) + \frac{2C^{\mathrm{r}}_{+}\widehat{G^{\mathrm{r}}}}{\varepsilon_{0}}, \tag{24}$$

$$R^{\mathrm{r}}_{-} \propto \frac{\omega^{\mathrm{r}}}{2}\left(\widehat{\alpha^{\mathrm{r}}}\left|\widetilde{\mathbf{E}^{\mathrm{r}}}\right|^{2} + T_{-} + \widehat{\chi^{\mathrm{r}}}\left|\widetilde{\mathbf{B}^{\mathrm{r}}}\right|^{2}\right) + \frac{2C^{\mathrm{r}}_{-}\widehat{G^{\mathrm{r}}}}{\varepsilon_{0}}. \tag{25}$$

Then, the ROA intensity is found to be

$$R^{\mathrm{e}}_{+}R^{\mathrm{r}}_{+} - R^{\mathrm{e}}_{-}R^{\mathrm{r}}_{-} \propto \left[\frac{\omega^{\mathrm{e}}}{2}\left(\widehat{\alpha^{\mathrm{e}}}\left|\widetilde{\mathbf{E}^{\mathrm{e}}}\right|^{2} + T^{\mathrm{e}}_{\mathrm{s}} + \widehat{\chi^{\mathrm{e}}}\left|\widetilde{\mathbf{B}^{\mathrm{e}}}\right|^{2}\right) + \frac{\omega^{\mathrm{e}}T^{\mathrm{e}}_{\mathrm{as}}}{2} + \frac{2C^{\mathrm{e}}\widehat{G^{\mathrm{e}}}}{\varepsilon_{0}}\right]\left[\frac{\omega^{\mathrm{r}}}{2}\left(\widehat{\alpha^{\mathrm{r}}}\left|\widetilde{\mathbf{E}^{\mathrm{r}}}\right|^{2} + T_{+} + \widehat{\chi^{\mathrm{r}}}\left|\widetilde{\mathbf{B}^{\mathrm{r}}}\right|^{2}\right) + \frac{2C^{\mathrm{r}}_{+}\widehat{G^{\mathrm{r}}}}{\varepsilon_{0}}\right] -$$

$$\left[\frac{\omega^{\mathrm{e}}}{2}\left(\widehat{\alpha^{\mathrm{e}}}\left|\widetilde{\mathbf{E}^{\mathrm{e}}}\right|^{2} + T^{\mathrm{e}}_{\mathrm{s}} + \widehat{\chi^{\mathrm{e}}}\left|\widetilde{\mathbf{B}^{\mathrm{e}}}\right|^{2}\right) - \frac{\omega^{\mathrm{e}}T^{\mathrm{e}}_{\mathrm{as}}}{2} - \frac{2C^{\mathrm{e}}\widehat{G^{\mathrm{e}}}}{\varepsilon_{0}}\right]\left[\frac{\omega^{\mathrm{r}}}{2}\left(\widehat{\alpha^{\mathrm{r}}}\left|\widetilde{\mathbf{E}^{\mathrm{r}}}\right|^{2} + T_{-} + \widehat{\chi^{\mathrm{r}}}\left|\widetilde{\mathbf{B}^{\mathrm{r}}}\right|^{2}\right) + \frac{2C^{\mathrm{r}}_{-}\widehat{G^{\mathrm{r}}}}{\varepsilon_{0}}\right]. \tag{26}$$

To understand the underlying physics of chiral-field-enhanced ROA, we need to consider Eq. (26) under some approximations for simplification. For most chiral molecules in a chiral field with a strong enhancement of optical chirality, especially for those composed of idealized axially symmetric bonds[5], the signal intensity contribution from the electric-field gradient ωT is much smaller than that from the optical chirality $2C\hat{G}/\varepsilon_{0}$. Moreover, for non-magnetic chiral molecules, $\hat{\chi}\left|\widetilde{\mathbf{B}}\right|^{2}$ is much smaller than $\hat{\alpha}\left|\widetilde{\mathbf{E}}\right|^{2}$. Thus, Eq. (26) can be simplified to

$$R^{\mathrm{e}}_{+}R^{\mathrm{r}}_{+} - R^{\mathrm{e}}_{-}R^{\mathrm{r}}_{-} \propto \left[\frac{\omega^{\mathrm{e}}\widehat{\alpha^{\mathrm{e}}}\left|\widetilde{\mathbf{E}^{\mathrm{e}}}\right|^{2}}{2} + \frac{2C^{\mathrm{e}}\widehat{G^{\mathrm{e}}}}{\varepsilon_{0}}\right]\left[\frac{\omega^{\mathrm{r}}\widehat{\alpha^{\mathrm{r}}}\left|\widetilde{\mathbf{E}^{\mathrm{r}}}\right|^{2}}{2} + \frac{2C^{\mathrm{r}}_{+}\widehat{G^{\mathrm{r}}}}{\varepsilon_{0}}\right] - \left[\frac{\omega^{\mathrm{e}}\widehat{\alpha^{\mathrm{e}}}\left|\widetilde{\mathbf{E}^{\mathrm{e}}}\right|^{2}}{2} - \frac{2C^{\mathrm{e}}\widehat{G^{\mathrm{e}}}}{\varepsilon_{0}}\right]\left[\frac{\omega^{\mathrm{r}}\widehat{\alpha^{\mathrm{r}}}\left|\widetilde{\mathbf{E}^{\mathrm{r}}}\right|^{2}}{2} + \frac{2C^{\mathrm{r}}_{-}\widehat{G^{\mathrm{r}}}}{\varepsilon_{0}}\right]. \tag{27}$$

If we expand the right-hand side of the equation, it can be expressed as

$$R^{\mathrm{e}}_{+}R^{\mathrm{r}}_{+} - R^{\mathrm{e}}_{-}R^{\mathrm{r}}_{-} \propto 2\frac{2C^{\mathrm{e}}\widehat{G^{\mathrm{e}}}}{\varepsilon_{0}}\frac{\omega^{\mathrm{r}}\widehat{\alpha^{\mathrm{r}}}\left|\widetilde{\mathbf{E}^{\mathrm{r}}}\right|^{2}}{2} + \frac{\omega^{\mathrm{e}}\widehat{\alpha^{\mathrm{e}}}\left|\widetilde{\mathbf{E}^{\mathrm{e}}}\right|^{2}}{2}\left[\frac{2C^{\mathrm{r}}_{+}\widehat{G^{\mathrm{r}}}}{\varepsilon_{0}} - \frac{2C^{\mathrm{r}}_{-}\widehat{G^{\mathrm{r}}}}{\varepsilon_{0}}\right] + \frac{2C^{\mathrm{e}}\widehat{G^{\mathrm{e}}}}{\varepsilon_{0}}\left[\frac{2C^{\mathrm{r}}_{+}\widehat{G^{\mathrm{r}}}}{\varepsilon_{0}} + \frac{2C^{\mathrm{r}}_{-}\widehat{G^{\mathrm{r}}}}{\varepsilon_{0}}\right]. \tag{28}$$

This equation can be approximated as

$$R^{\mathrm{e}}_{+}R^{\mathrm{r}}_{+} - R^{\mathrm{e}}_{-}R^{\mathrm{r}}_{-} \propto 2\frac{2C^{\mathrm{e}}\widehat{G^{\mathrm{e}}}}{\varepsilon_{0}}\frac{\omega^{\mathrm{r}}\widehat{\alpha^{\mathrm{r}}}\left|\widetilde{\mathbf{E}^{\mathrm{r}}}\right|^{2}}{2} + \frac{\omega^{\mathrm{e}}\widehat{\alpha^{\mathrm{e}}}\left|\widetilde{\mathbf{E}^{\mathrm{e}}}\right|^{2}}{2}\left[\frac{2C^{\mathrm{r}}_{+}\widehat{G^{\mathrm{r}}}}{\varepsilon_{0}} - \frac{2C^{\mathrm{r}}_{-}\widehat{G^{\mathrm{r}}}}{\varepsilon_{0}}\right], \tag{29}$$

where we have used $\frac{2C\hat{G}}{\varepsilon_{0}} \ll \frac{\omega\hat{\alpha}\left|\widetilde{\mathbf{E}}\right|^{2}}{2}$. To verify that this condition for the approximation is valid, we reconsider Eq. (15), which relates C with $\widetilde{\mathbf{E}}$. For circularly polarized light, we obtain from Eq. (15)

$$C = \frac{\omega\varepsilon_0|\tilde{\mathbf{E}}|^2}{2c}. \tag{30}$$

In addition, we have $|\tilde{\mathbf{B}}| = \frac{|\tilde{\mathbf{E}}|}{c}$ and $\hat{G}|\tilde{\mathbf{B}}| \ll \hat{\alpha}|\tilde{\mathbf{E}}|$ from Eq. (6) as $\hat{G}|\tilde{\mathbf{B}}|$ is a high-order term. Thus, we have $\hat{G} \ll c\hat{\alpha}$. By simultaneously multiplying both the right- and left-hand sides of this relation by $\frac{2C}{\varepsilon_0}$ (where C is positive based on our definition), we have

$$\frac{2C\hat{G}}{\varepsilon_0} \ll \frac{2Cc\hat{\alpha}}{\varepsilon_0}. \tag{31}$$

By substituting Eq. (30) into the right-hand side of Eq. (31), we have

$$\frac{2C\hat{G}}{\varepsilon_0} \ll \frac{\omega\hat{\alpha}|\tilde{\mathbf{E}}|^2}{2}. \tag{32}$$

Therefore, with this condition, $\frac{2C^{\mathrm{e}}\widehat{G^{\mathrm{e}}}}{\varepsilon_0}\left[\frac{2C_+^{\mathrm{r}}\widehat{G^{\mathrm{r}}}}{\varepsilon_0} + \frac{2C_-^{\mathrm{r}}\widehat{G^{\mathrm{r}}}}{\varepsilon_0}\right]$ in Eq. (28) is a high-order term and can be omitted. The first and second terms on the right-hand side of Eq. (29) correspond to incident circular polarization ROA (ICP-ROA) and scattered circular polarization ROA (SCP-ROA), respectively. For ICP-ROA, the optical chirality (circular polarization) of incident light (excitation field) C^{e} is well controlled while the optical chirality of Raman scattering (radiation field) C^{r} needs to be scrambled[3]. This means that the circularly polarized components of Raman scattering was eliminated in our measurement, which corresponds to $\frac{\omega^{\mathrm{e}}\widehat{\alpha^{\mathrm{e}}}|\tilde{\mathbf{E}}^{\mathrm{e}}|^2}{2}\left[\frac{2C_+^{\mathrm{r}}\widehat{G^{\mathrm{r}}}}{\varepsilon_0} - \frac{2C_-^{\mathrm{r}}\widehat{G^{\mathrm{r}}}}{\varepsilon_0}\right] = 0$. It is noted that, for all ICP-ROA measurements, one measures the scattered Raman intensities with either no polarization or some specified state of linear polarization without any circular or elliptical polarization content[3]. Moreover, the silicon nanodisk array also scrambles the optical chirality of Raman scattering as it cannot enhance but even reduce the optical chirality from the near field to the far field at the Raman scattering wavelength (Supplementary Fig. 1) by considering optical reciprocity. For SCP-ROA, the optical chirality (circular polarization) of incident light (excitation field) C^{e} needs to be scrambled while the optical chirality of Raman scattering (radiation field) C^{r} is measured[3]. This means the circularly polarized components of incident light was eliminated in the measurement, which corresponds to $2\frac{2C^{\mathrm{e}}\widehat{G^{\mathrm{e}}}}{\varepsilon_0}\frac{\omega^{\mathrm{r}}\widehat{\alpha^{\mathrm{r}}}|\tilde{\mathbf{E}}^{\mathrm{r}}|^2}{2} = 0$. As we used ICP-ROA in our experiment, $\frac{\omega^{\mathrm{e}}\widehat{\alpha^{\mathrm{e}}}|\tilde{\mathbf{E}}^{\mathrm{e}}|^2}{2}\left[\frac{2C_+^{\mathrm{r}}\widehat{G^{\mathrm{r}}}}{\varepsilon_0} - \frac{2C_-^{\mathrm{r}}\widehat{G^{\mathrm{r}}}}{\varepsilon_0}\right]$ in Eq. (32) can be omitted in our measurement. The measured ICP-ROA intensity can be rewritten as

$$R_+^{\mathrm{e}}R_+^{\mathrm{r}} - R_-^{\mathrm{e}}R_-^{\mathrm{r}} \propto \frac{2\omega^{\mathrm{r}}\widehat{\alpha^{\mathrm{r}}}\widehat{G^{\mathrm{e}}}C^{\mathrm{e}}\left|\widehat{\mathbf{E}^{\mathrm{r}}}\right|^2}{\varepsilon_0}, \tag{33}$$

which means that C^{r} is negligible for our ICP-ROA measurement. Therefore, Eq. (27) can be rewritten as

$$R_+^{\mathrm{e}}R_+^{\mathrm{r}} - R_-^{\mathrm{e}}R_-^{\mathrm{r}} \propto \left[\frac{\omega^{\mathrm{e}}\widehat{\alpha^{\mathrm{e}}}\left|\widehat{\mathbf{E}^{\mathrm{e}}}\right|^2}{2} + \frac{2C^{\mathrm{e}}\widehat{G^{\mathrm{e}}}}{\varepsilon_0}\right]\left[\frac{\omega^{\mathrm{r}}\widehat{\alpha^{\mathrm{r}}}\left|\widehat{\mathbf{E}^{\mathrm{r}}}\right|^2}{2}\right] - \left[\frac{\omega^{\mathrm{e}}\widehat{\alpha^{\mathrm{e}}}\left|\widehat{\mathbf{E}^{\mathrm{e}}}\right|^2}{2} - \frac{2C^{\mathrm{e}}\widehat{G^{\mathrm{e}}}}{\varepsilon_0}\right]\left[\frac{\omega^{\mathrm{r}}\widehat{\alpha^{\mathrm{r}}}\left|\widehat{\mathbf{E}^{\mathrm{r}}}\right|^2}{2}\right] = \frac{2\omega^{\mathrm{r}}\widehat{\alpha^{\mathrm{r}}}\widehat{G^{\mathrm{e}}}C^{\mathrm{e}}\left|\widehat{\mathbf{E}^{\mathrm{r}}}\right|^2}{\varepsilon_0}. \tag{34}$$

Based on Eq. (34), the circular intensity difference (CID) of the ROA can be expressed as

$$\mathrm{CID} = \frac{I_{\mathrm{RCP}} - I_{\mathrm{LCP}}}{I_{\mathrm{RCP}} + I_{\mathrm{LCP}}} \propto \frac{R_+^{\mathrm{e}}R_+^{\mathrm{r}} - R_-^{\mathrm{e}}R_-^{\mathrm{r}}}{R_+^{\mathrm{e}}R_+^{\mathrm{r}} + R_-^{\mathrm{e}}R_-^{\mathrm{r}}} \propto \frac{8\widehat{G^{\mathrm{e}}}C^{\mathrm{e}}}{\varepsilon_0\omega^{\mathrm{e}}\widehat{\alpha^{\mathrm{e}}}\left|\widehat{\mathbf{E}^{\mathrm{e}}}\right|^2}, \tag{35}$$

where I_{RCP} and I_{LCP} are the intensities of Raman scattering excited by incident RCP and LCP light, respectively.

Supplementary Note 4: Fabrication of the silicon nanodisk array

Steps for fabricating the silicon nanodisk array are schematically illustrated in Fig. 2a. Firstly, a 130-nm-thick HSQ negative resist film was spin-coated on a silicon-on-insulator chip whose top silicon layer thickness is 180 nm. Then, the nanodisk array pattern was defined on the resist film by electron beam lithography (EBL) with an exposure dose of 3500 μC/cm^2. After that, the chip was developed in an NMD-3 solution for 20 minutes and rinsed in deionized water for 5 minutes. The nanodisk array pattern was transferred to the top silicon layer via inductively coupled plasma by using a mixed gas of O_2 and SF_6 at a temperature of -70°C. Finally, the remaining HSQ resist was removed by using the diluted hydrofluoric acid with a concentration of 1%.

Supplementary Note 5: Optical characterization of the silicon nanodisk array

The reflection spectra of the silicon nanodisk array were obtained by using a home-made optical spectroscopy setup as shown in Supplementary Fig. 2. We used a broadband Tungsten-Halogen light source (Thorlabs SLS201L) to provide excitation light. The circular polarization state of the excitation light was controlled by using a linear polarizer and an achromatic quarter-wave plate. To maintain the polarization state of the excitation light, a low numerical aperture objective lens (Olympus UPLSAPO 10× 0.40 N.A.) was used to focus the excitation light on the silicon nanodisk array. The silicon nanodisk array chip was immersed in a cuvette filled with a mixed solution of 50% (+)-alpha-pinene and 50% (-)-alpha-pinene, which provided an effective achiral

refractive-index environment. The reflected light from the silicon nanodisk array was collected by the same objective lens and was then split into two beams by a beam splitter. One beam was used to image and locate the silicon nanodisk array on a CMOS camera via a lens. The other beam was coupled to a spectrometer via a lens to measure the reflection spectra of the silicon nanodisk array.

Supplementary Note 6: ROA measurement by using a home-made two-phase virtual-enantiomer ROA setup

We measured the ROA signals with the home-made two-phase virtual-enantiomer ROA setup as shown in Fig. 2c. The optical setup is based on an incident circularly polarized ROA configuration[4]. A continuous-wave laser with an emission wavelength of 532 nm was used as the light source. The circular polarization state of the output light was realized by using a linear polarizer and a 532-nm zeroth-order quarter-wave plate. A 532-nm zeroth-order half-wave plate mounted in a motorized flipper was used to modulate the circular polarization state of the output light between the LCP and RCP states by placing it in and out of the optical path. The modulated light passed through the hole in an off-axis parabolic mirror and was focused on samples on the silicon nanodisk array chip via an optical lens. Another 532-nm zeroth-order half-wave plate mounted in a motorized flipper was placed between the off-axis parabolic mirror and the optical lens to provide the configuration for two-phase virtual-enantiomer measurement. In Phase I in which the half-wave plate was moved out of the optical path, the measured ROA signals reflected the sample's original chirality. In Phase II in which the half-wave plate was moved into the optical path, the measured sample with the half-wave plate as a whole was virtually converted to the enantiomer of the sample. By subtracting the measured ROA signals in Phase II from those in Phase I, the artifacts due to the systematic offset in the optical setup was suppressed. The two-phase modulation period was 40 times larger than the modulation period of the incident circularly polarized state. After that, the back-scattered light from the samples was collected by the same optical lens and reflected by the off-axis parabolic mirror. The reflected light then passed through a notch filter that filtered its Rayleigh scattering component and a 561-nm rotating zero-order half-wave plate that averaged its linear polarizations to zero over time. Finally, the remaining light entered a spectrometer with a cooled charge-coupled device detector where the Raman spectra of the samples were measured. The ROA signals were obtained by subtracting the accumulative Raman spectra excited

by the incident LCP light from those excited by the incident RCP light. All the optical components and the measured data were controlled, synchronized, tracked, and saved by using NI Labview software. Additionally, there was another assistant optical branch used to locate on the chip, mainly comprising a halogen lamp for illumination and a CMOS camera for imaging. The two beam splitters were used to guide the illumination light for imaging, which could be moved out from the optical path for ROA measurements.

Supplementary Note 7: Raman and ROA enhancement factors achieved by the silicon nanodisk array

To quantify the Raman and ROA enhancement factors achieved by the silicon nanodisk array, we picked up all the Raman and ROA peaks of (±)-alpha-pinene in Fig. 3a and Fig. 3b and calculated their enhancement factors. The results of our direct measurements that include the near-field and far-field contributions to the Raman and ROA spectra are shown in Supplementary Fig. 3. For (±)-tartaric-acid, the results are shown in Supplementary Fig. 5. With linear fitting shown in Supplementary Fig. 4, an average Raman signal ratio between the silica substrate and silicon nanodisk array was found to be 1.65 while an average ROA signal ratio between the silica substrate and silicon nanodisk array was found to be 1.61. The similar average signal ratios for the Raman and ROA signals means that the CID value for the enhanced ROA signal is similar to the CID value for the unenhanced ROA signal. This indicates an efficient conversion of the optical chirality of the incident light to the near field for enhanced ROA excitation, which agrees well with our analysis in Supplementary Section 9. The signal ratios were obtained by measuring 25-μm-thick (±)-alpha-pinene layers prepared by immersing our 725-μm-thick chip into a cuvette with an optical path length of 0.75 mm. It is important to note that it is technically difficult to prepare an ultrathin alpha-pinene (volatile liquid) film with the thickness of the near-field region (~250 nm) that is perfectly sealed on our chip and does not evaporate in the long-time measurement (several hours) for directly measuring the near-field Raman and ROA signals. Based on the above experimentally measured signal ratios, their enhancement factors in the near-field region can be estimated by using the following equation:

$$\frac{(V_\mathrm{n}-V_\mathrm{d})\mathrm{EF}+V_\mathrm{t}-V_\mathrm{n}}{V_\mathrm{t}-V_\mathrm{d}}=R_\mathrm{s}, \tag{36}$$

where V_n is the volume of the near-field region, V_d is the volume of the silicon nanodisk array in the near-field

region, V_t is the total volume of the sample, EF is the enhancement factor in the near-field region, R_s is the signal ratio for the total volume of the sample between the two substrates. In our case, based on the electric-field distribution, the near-field region for the silicon nanodisk array is a 250-nm-thick layer on the silica substrate. Thus, $V_\mathrm{n} = (0.25\ \mu\mathrm{m})S$, where S is the optical illumination area. As the height of the silicon nanodisk array is 180 nm while its filling factor in the layer is 0.577, $V_\mathrm{d} = (0.18\ \mu\mathrm{m})S \times 0.577 = (0.10\ \mu\mathrm{m})S$. For the 25-μm-thick samples, $V_\mathrm{t} = (25\ \mu\mathrm{m})S$. Based on the above values and $R_\mathrm{s} = 1.61$ for the average Raman signal ratio, the Raman enhancement factor EF in the near-field region is found to be 109. Similarly, the ROA enhancement factor EF in the near-field region is found to be 102. Therefore, the ROA enhancement factor in the near-field region achieved by the silicon nanodisk array is $\sim 10^2$. In order to compare the experimental results with the theoretical results, we calculated the theoretical Raman and ROA enhancement factors in the near-field region. Based on Eq. (35), the theoretical Raman enhancement factor at a specific point in the near-field region is found to be

$$\mathrm{EF_{Raman}} = \frac{I_\mathrm{RCP}+I_\mathrm{LCP}}{I_\mathrm{0RCP}+I_\mathrm{0LCP}} = \frac{R_+^\mathrm{e}R_+^\mathrm{r}+R_-^\mathrm{e}R_-^\mathrm{r}}{R_{0+}^\mathrm{e}R_{0+}^\mathrm{r}+R_{0-}^\mathrm{e}R_{0-}^\mathrm{r}} = \left|\frac{\widetilde{\mathbf{E}}^\mathrm{e}}{\widetilde{\mathbf{E}}_0^\mathrm{e}}\right|^2 \left|\frac{\widetilde{\mathbf{E}}^\mathrm{r}}{\widetilde{\mathbf{E}}_0^\mathrm{r}}\right|^2, \tag{37}$$

where the subscript 0 indicates the physical variables without the chiral-field enhancement. The theoretical Raman enhancement factor in the near-field region can be analytically calculated to be

$$\mathrm{EF_{Raman}} = \frac{\int_{V_\mathrm{d}}^{V_\mathrm{n}} \left|\frac{\widetilde{\mathbf{E}}^\mathrm{e}}{\widetilde{\mathbf{E}}_0^\mathrm{e}}\right|^2 \left|\frac{\widetilde{\mathbf{E}}^\mathrm{r}}{\widetilde{\mathbf{E}}_0^\mathrm{r}}\right|^2 dv}{\int_{V_\mathrm{d}}^{V_\mathrm{n}} dv}. \tag{38}$$

It is noted that $\left|\frac{\widetilde{\mathbf{E}}^\mathrm{r}}{\widetilde{\mathbf{E}}_0^\mathrm{r}}\right|^2$ which corresponds to the near-field enhancement is approximately equal to the far-field radiation enhancement at the excitation wavelength of Raman scattering due to the optical reciprocity theorem. Based on this equation, the theoretical Raman enhancement factor in the near-field region is estimated to be 93, which is on the order of $\sim 10^2$ and agrees with our experimental result. Similarly based on Eq. (34), the theoretical ROA enhancement factor at a specific point in the near-field region is found to be

$$\mathrm{EF_{ROA}} = \frac{I_\mathrm{RCP}-I_\mathrm{LCP}}{I_\mathrm{0RCP}-I_\mathrm{0LCP}} = \frac{R_+^\mathrm{e}R_+^\mathrm{r}-R_-^\mathrm{e}R_-^\mathrm{r}}{R_{0+}^\mathrm{e}R_{0+}^\mathrm{r}-R_{0-}^\mathrm{e}R_{0-}^\mathrm{r}} = \frac{C^\mathrm{e}}{C_0^\mathrm{e}} \left|\frac{\widetilde{\mathbf{E}}^\mathrm{r}}{\widetilde{\mathbf{E}}_0^\mathrm{r}}\right|^2. \tag{39}$$

It is evident from Eq. (39) that the silicon nanodisk array works as a chiral-field generator to enhance the chiral light-molecule interaction to enhance the Raman intensity difference between LCP and RCP excitation in the

excitation process while it works as an optical antenna to enhance the far-field radiation to further enhance the Raman intensity difference between LCP and RCP excitation in the radiation process. The theoretical ROA enhancement factor in the near-field region can be analytically calculated by

$$\mathrm{EF_{ROA}} = \frac{\int_{V_\mathrm{d}}^{V_\mathrm{n}} \frac{C^\mathrm{e}}{C_0^\mathrm{e}} \left|\frac{\widetilde{\mathbf{E}}^\mathrm{r}}{\widetilde{\mathbf{E}}_0^\mathrm{r}}\right|^2 dv}{\int_{V_\mathrm{d}}^{V_\mathrm{n}} dv}. \tag{40}$$

Based on this equation, the theoretical ROA enhancement factor in the near-field region is estimated to be 102, which is also on the order of ~10^2 and agrees with our experimental result. To further verify the theoretical ROA enhancement factor, we also evaluate the theoretical dissymmetric factor in the silicon nanodisk array, which determines the enhancement factor of the CID. As the estimated dissymmetric factor in the silicon nanodisk array is ~1 (Supplementary Note 8), the CID enhancement factor is ~1, which means the theoretical ROA enhancement factor is almost identical to the theoretical Raman enhancement factor, which also agrees well with the above result.

Supplementary Note 8: Dissymmetric factor achieved by the silicon nanodisk array

The dissymmetric factor indicates the strength of chiral light-matter interaction based on intensity-normalized light, which excludes the influence of light intensity by normalization. This parameter predominantly determines the CID of ROA measurements. Based on Eq. (35), we have

$$\mathrm{CID} = \frac{I_\mathrm{RCP} - I_\mathrm{LCP}}{I_\mathrm{RCP} + I_\mathrm{LCP}} \propto \frac{R_+^\mathrm{e} R_+^\mathrm{r} - R_-^\mathrm{e} R_-^\mathrm{r}}{R_+^\mathrm{e} R_+^\mathrm{r} + R_-^\mathrm{e} R_-^\mathrm{r}} \propto \frac{8\widehat{G}^\mathrm{e} C^\mathrm{e}}{\varepsilon_0 \omega^\mathrm{e} \widehat{\alpha}^\mathrm{e} \left|\widetilde{\mathbf{E}}^\mathrm{e}\right|^2} \propto g. \tag{41}$$

As $\widehat{G}^\mathrm{e}$, ε_0, $\widehat{\alpha}^\mathrm{e}$, and ω^e are constants for a molecule excited by light with a fixed frequency, the normalized circular excitation rate difference $\frac{R_+ - R_-}{R_+ + R_-}$ is determined by $C^\mathrm{e}/\left|\widetilde{\mathbf{E}}^\mathrm{e}\right|^2$ and ends up as the dissymmetric factor g. Therefore, the dissymmetric factor g is the optical chirality normalized to the light intensity, which is a parameter that quantifies the chiral light-matter interaction strength by the intensity-normalized incident light. Based on the above analysis, with incident RCP light as an example, we calculated the distribution of dissymmetric factor $g/|g_\mathrm{CPL}|$, which is normalized to the dissymmetric factor of the free-space circularly polarized light in a unit cell of the silicon nanodisk array. The result is shown in Supplementary Fig. 6. It is evident that even though the incident circularly light is converted and confined to the near field, its structure for

chiral light-matter interaction is not decayed. We further calculated the average dissymmetric factor in the near-field region by using

$$g_{\mathrm{a}} = \frac{\int_{V_{\mathrm{d}}}^{V_{\mathrm{n}}} g(x,y,z)/|g_{\mathrm{CPL}}| dv}{\int_{V_{\mathrm{d}}}^{V_{\mathrm{n}}} dv}. \quad (42)$$

From the equation, we obtained $g_{\mathrm{a}} = 1.3$, which is on the order of ~1. Thus, the normalized circular excitation rate difference of a chiral molecule in the near-field region of the silicon nanodisk array is similar to that in the region illuminated by the free-space circularly polarized light. Therefore, the CID of the chiral-field enhanced ROA by the silicon nanodisk array should be similar to that without the enhancement, which agrees well with our experimental results. Additionally, this g_{a} value of the silicon nanodisk array indicates its high efficiency for the optical-chirality transfer from the far field to the near field by using the dark mode.

Supplementary Note 9: Photothermal heating in the silicon nanodisk array

To show low photothermal heating in the silicon nanodisk array in comparison with its plasmonic counterpart, we used the finite element method (FEM) to simulate temperature distributions of both the silicon nanodisk array and its plasmonic counterpart with an identical incident power density of 5 mW/μm^2 in a thermal equilibrium state. The wavelength of the incident light is 532 nm while the environmental temperature is 25 °C. The plasmonic counterpart has an identical geometric structure to that of the silicon nanodisk array, but is made of silver. It is evident from the simulation result shown in Supplementary Fig. 7 that the silicon nanodisk array has a lower temperature than its plasmonic counterpart under the identical experimental conditions, which verifies the relatively low photothermal heat generation in the silicon nanodisk array.

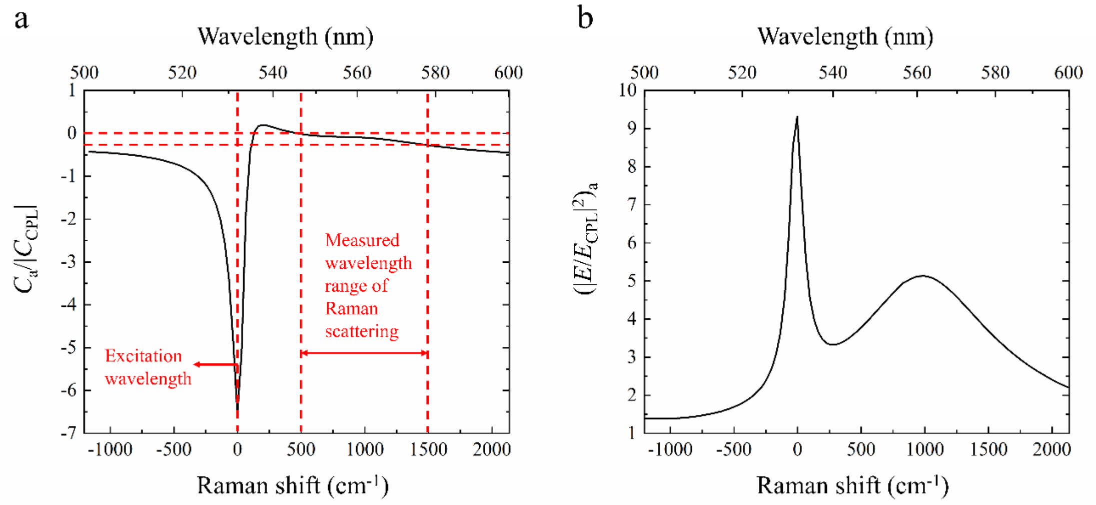

Supplementary Fig. 1 | Effects in the near-field region. a, Average optical activity (RCP excitation) in the near-field region on the silicon nanodisk array as a function of wavelength (Raman shift), normalized by the optical chirality of incident CPL in the far field. **b,** Averaged square of the electric-field magnitude in the near-field region on the silicon nanodisk array as a function of wavelength (Raman shift), normalized by the square of the electric-field magnitude of incident CPL in the far field.

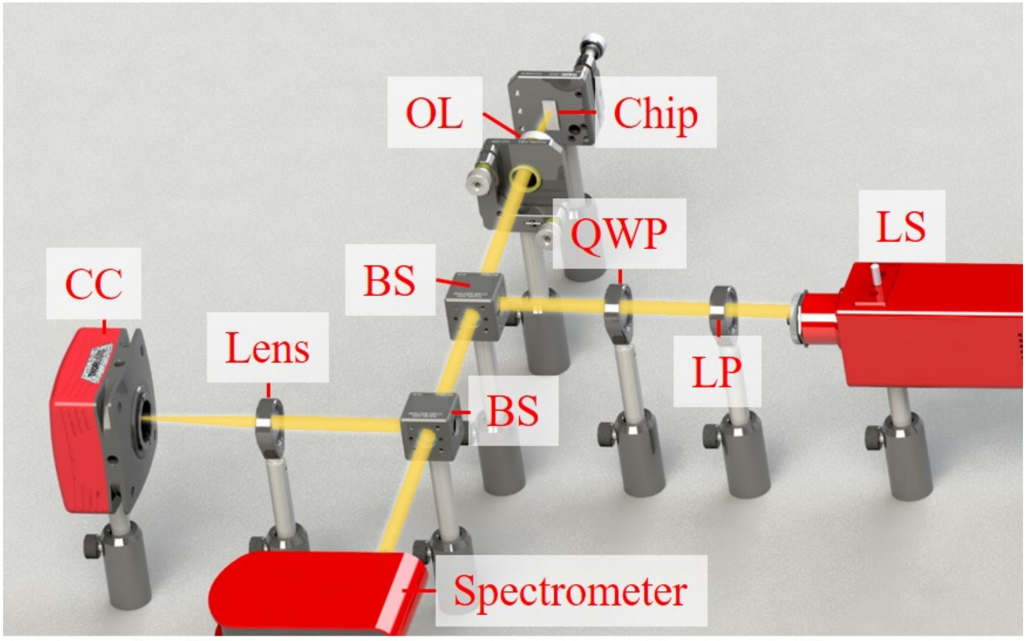

Supplementary Fig. 2 | Schematic of the reflection spectroscopy setup. OL: Objective lens. QWP: Quarter-wave plate. BS: Beam splitter. LS: Light source. LP: Linear polarizer. CC: CMOS camera.

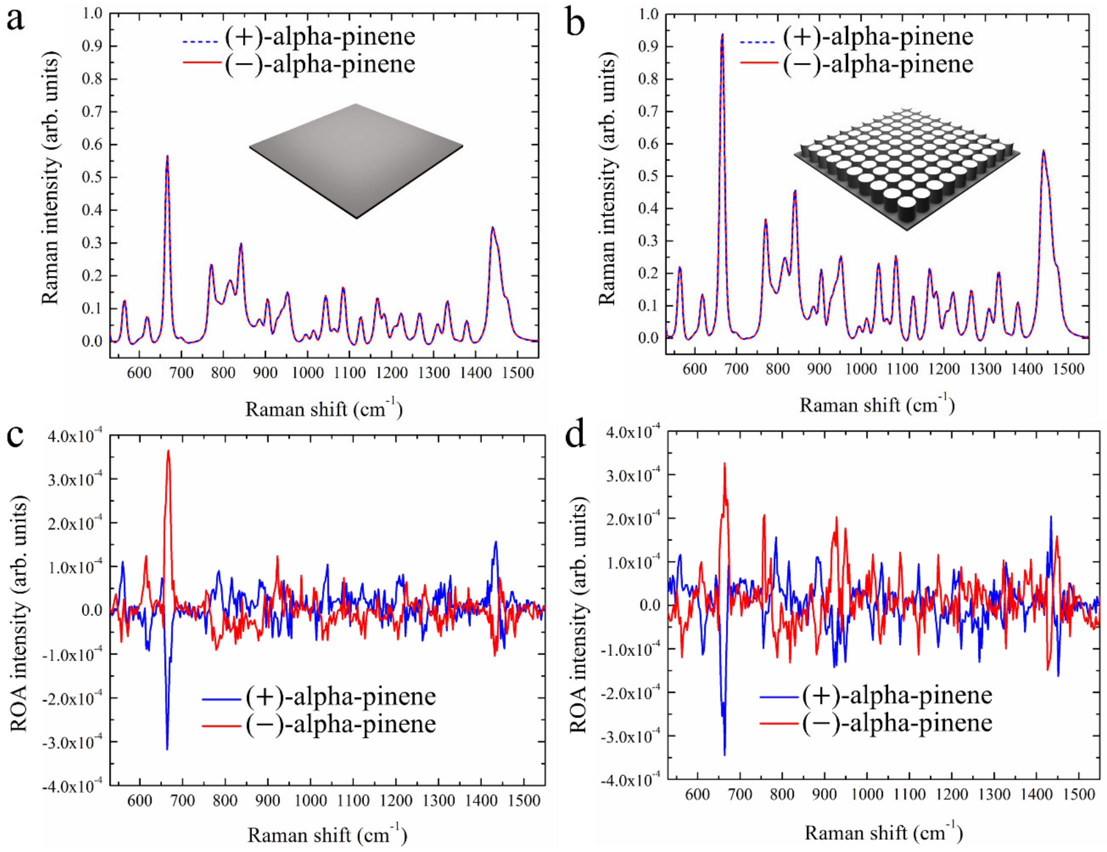

Supplementary Fig. 3 | Measured Raman and ROA spectra of (±)-alpha-pinene. a, Raman spectra of (±)-alpha-pinene on the silica substrate. **b,** Raman spectra of (±)-alpha-pinene on the silicon nanodisk array. **c**, ROA spectra of (±)-alpha-pinene on the silica substrate. **d,** ROA spectra of (±)-alpha-pinene on the silicon nanodisk array.

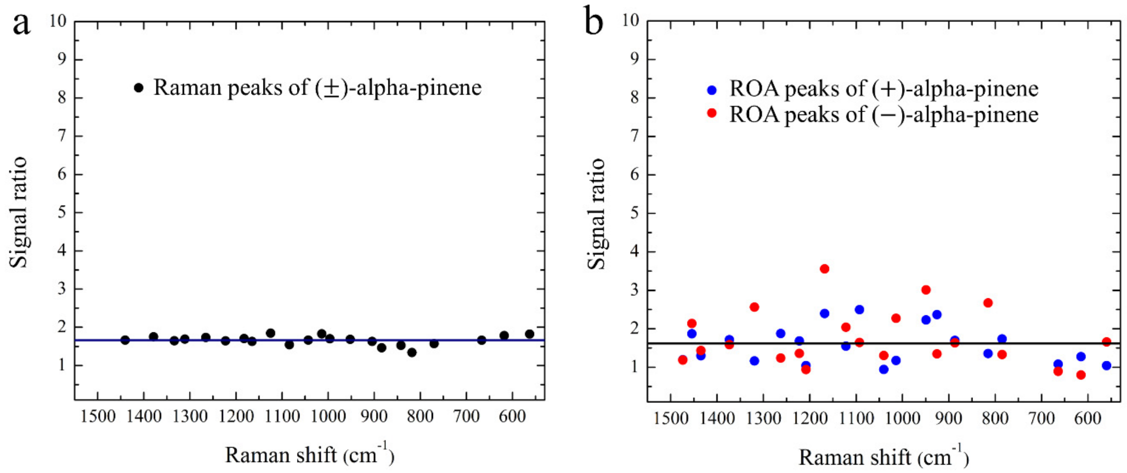

Supplementary Fig. 4 | Experimentally measured Raman and ROA signal ratios of (±)-alpha-pinene between the silica substrate and silicon nanodisk array. a, Ratios of the Raman peaks of (±)-alpha-pinene. **b,** Ratios of the ROA peaks of (±)-alpha-pinene.

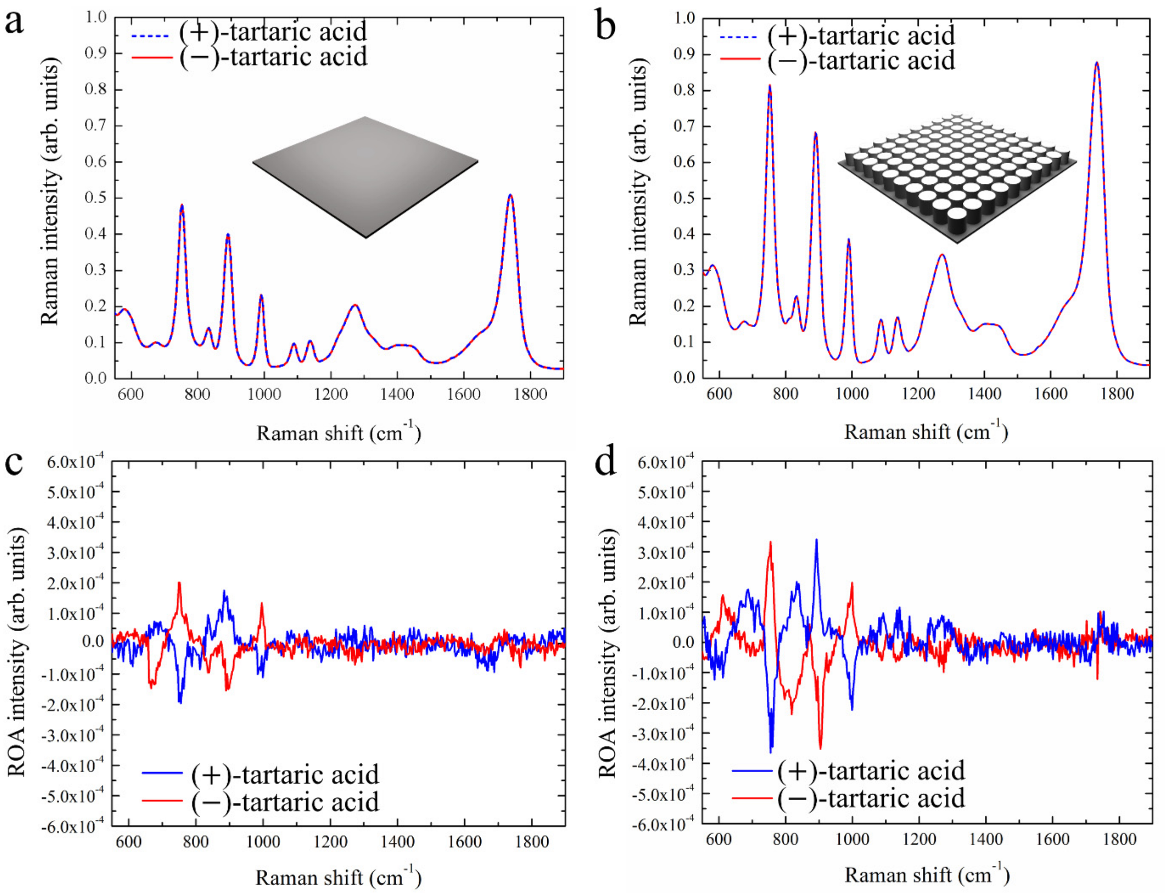

Supplementary Fig. 5 | Measured Raman and ROA spectra of (±)-tartaric acid. a, Raman spectra of (±)-tartaric acid on the silica substrate. **b,** Raman spectra of (±)-tartaric acid on the silicon nanodisk array. **c**, ROA spectra of (±)-tartaric acid on the silica substrate. **d,** ROA spectra of (±)-tartaric acid on the silicon nanodisk array.

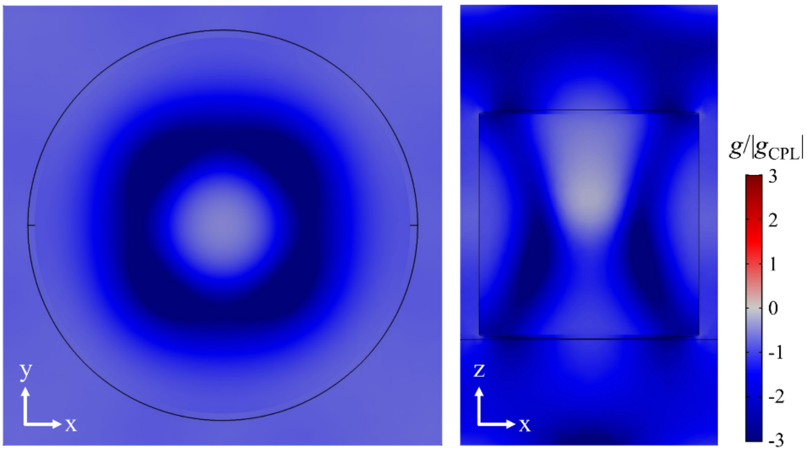

Supplementary Fig. 6 | Dissymmetric factor distribution in a unit cell of the silicon nanodisk array.

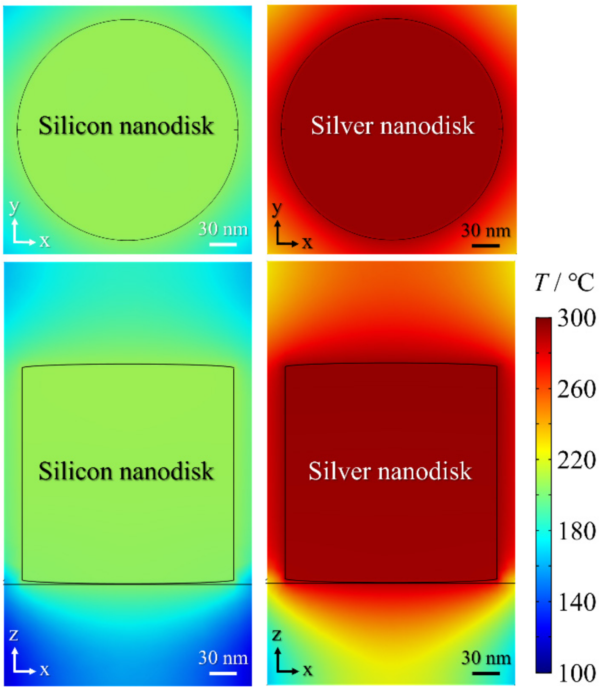

Supplementary Fig. 7 | Temperature distributions of the silicon nanodisk array and its plasmonic counterpart with an identical incident power density of 5 mW/μm^2 in a thermal equilibrium state.

Supplementary References

1. Vuye, G. et al. Temperature-dependence of the dielectric function of silicon using in-situ spectroscopic ellipsometry. *Thin Solid Films* **233,** 166-170 (1993).
2. Miroshnichenko, A. E. et al. Nonradiating anapole modes in dielectric nanoparticles. *Nat. Commun.* **6,** 8069 (2015).
3. Nafie, L. A. *Vibrational optical activity: principles and applications,* (John Wiley & Sons Ltd. West Sussex, 2011)
4. Yamamoto, S. et al. Incident circularly polarized Raman optical activity spectrometer based on circularity conversion method. *J. Raman Spectrosc.* **41,** 1664-1669 (2010).
5. Qiu, S. Chirality transition in the epoxidation of (-)-α-pinene and successive hydrolysis studied by Raman optical activity and DFT. *Phys. Chem. Chem. Phys.* **12,** 3005-3013 (2020)
